# Fc-apelin fusion protein attenuates lipopolysaccharide-induced liver injury in mice

**DOI:** 10.1038/s41598-018-29491-7

**Published:** 2018-07-30

**Authors:** Huifen Zhou, Rongze Yang, Weimin Wang, Feng Xu, Yue Xi, Robert A. Brown, Hong Zhang, Lin Shi, Dalong Zhu, Da-Wei Gong

**Affiliations:** 1 Department of Pathology, Hubei University of Science and Technology, Xianning, Hubei 437100 China; 2 Division of Endocrinology, Diabetes and Nutrition, University of Maryland School of Medicine, Baltimore, USA; 3 Nanjing University School of Medicine, Nanjing, China

## Abstract

Apelin is a peptide hormone with anti-oxidative and anti-inflammatory activities and is proposed to be a potential therapeutic for many disease conditions, including sepsis. However, short *in vivo* half-life of the apelin peptide would limit its potential clinical applications. This study aims to investigate the effects of Fc-apelin, a novel long-acting apelin fusion protein, on lipopolysaccharide (LPS)-induced liver injury. Liver injury was induced by systemic injection of LPS in mice. Hepatoprotective activities of Fc-apelin against inflammation were evaluated in LPS mice and/or hepatoma Huh-7 cells with respect to serum ALT, apoptosis, oxidative stress, macrophage infiltration and gene expression. We found that LPS induced systemic inflammation and liver damage. Co-administration of Fc-apelin significantly attenuated serum ALT elevation, diminished LPS-induced apoptosis and ROS production in the liver and in Huh-7 cells, mitigated hepatic macrophage infiltration, and reduced TNFα and IL-6 gene expression. Collectively, Fc-apelin fusion protein exerts protective effects against LPS-induced liver damage and may serve as a potential therapeutic for endotoxin-induced liver injury.

## Introduction

Endotoxemia is a common cause of multiple organ failure in the clinic. Hepatic injury by endotoxemia occurs early in multiorgan failure syndrome and is associated with poor prognosis. Despite advances in critical care, treatment for endotoxin-induced liver damage remains a challenge in clinical practice due to limited therapeutics. LPS-induced liver injury is a commonly-used model for mechanistic and therapeutic studies of endotoxemia-induced liver injury. Decades of research have revealed that activation of inflammatory pathways is the major mechanism for LPS-induced liver injury. One prevailing mechanism is that endotoxin activates macrophages in the liver^1–3^, which recruit and activate neutrophils, leading to over-production of reactive oxygen species (ROS) and oxidative stress in the liver^4,5^. Overproduction of ROS and small-scale inflammation induce transcription factor nuclear factor-кB (NF-кB) and subsequently results in a large scale inflammatory response and production of proinflammatory cytokines and chemokines^6,7^, leading to cell death by necrosis, apoptosis, and functional deterioration^1^.

Accordingly, suppression of oxidative stress and inflammation is found to alleviate LPS-induced liver injury. For example, ROS clearance and suppression of NF-κB alleviate liver injury^8–10^ in animals. However, clinical remedies for endotoxin-induced liver injury remain limited.

Apelin is a peptide hormone and ligand of the apelin receptor (APJ), a 7-transmembrane G protein-coupled receptor^11,12^. Numerous studies have demonstrated that apelin is a multifunctional hormone. Besides its inotropic activity, apelin has been reported to be anti-oxidative^13–15^. In addition, apelin can reduce inflammation and fibrosis and inhibit NF-κB activity in murine pancreatitis^16^, Moreover, apelin is known to activate PI3K/AKT/ERK signaling pathways and to promote cell survival^17,18^. We thus hypothesized that enhancement of apelin signaling would counteract LPS-mediated hepatic injury. Yet, apelin is a small peptide and has a very short *in vivo* half-life of about 8 mins^19,20^, which wound limit its clinical application^21^. To prolong its half-life, we engineered an apelin fusion protein by conjugating the Fc-fragment of human IgG with the apelin-13 peptide, resulting in Fc-apelin-13 (abbreviated as Fc-apelin hereafter)^22^. The fusion protein has an extended *in vivo* half-life of about 33 hours and has shown beneficial effects of improving insulin sensitivity and cardiac output in obese mice. In this report, we investigated whether Fc-apelin-13 fusion protein had protective effects against LPS-induced liver injury in mice.

## Materials and Methods

### Fc-apelin fusion protein

The production of Fc-apelin fusion protein is described previously^22^. In brief, the human IgG Fc-region is conjugated with apelin-13 at the N-terminus through the 3x linker of [Gly-Gly-Gly-Gly-Ser (GGGGS)]^23^. The protein is expressed in HEK293 cells and purified into homogeneity (>95% purity) through protein A affinity chromatography. Through buffer exchange, the fusion protein is dissolved in 1x PBS buffer, sterilized by filtering through a 0.02 μm filter, and stored at −20 °C until use.

### Animal studies

Animal experimental protocols were approved by the Institutional Animal Care and Use Committee of the University of Maryland and all methods were performed in accordance with the relevant guidelines and regulations. C57/BL6 female mice, 6 weeks old, were obtained from Charles River Laboratories (Wilmington, MA) and used after one week of quarantine and acclimatization. Experimental animals were randomly divided into four groups according to the treatment they received: (1) control (Cont), receiving injection of vehicle PBS intraperitoneally (i.p.) and subcutaneously (s.c.); (2) Fc-apelin, receiving Fc-apelin (1 mg/kg/day), s.c. and PBS, i.p.; (3) LPS-challenged group (LPS), receiving LPS (1 mg/kg) i.p and PBS, s.c.; and (4); LPS/Fc-apelin group, receiving LPS (1 mg/kg), i.p. and Fc-apelin (1 mg/kg), s.c. Mice were administered PBS, LPS and/or Fc-apelin daily for five consecutive days. Six hours after final injection, the mice were sacrificed. Blood samples were collected, and sera separated and stored at −20 °C until analysis. Depending on the application, liver specimens were embedded in optimal cutting temperature compound Tissue-Teck (Sakura, CA), fixed in 10% neutral buffered formalin for histological analysis, or snap frozen in liquid nitrogen.

### Serum ALT and IL-6 assay

Serum alanine transaminase (ALT) was measured by using the DiscretPak ALT substrate reagent (Catachem, Bridgeport, CT) and interleukin-6 (IL-6) levels were measured by ELISA kit (eBioscience, San Diego, CA), as described before^24,25^.

### Cell culture

Human Huh-7 hepatoma cells obtained from the American Type Culture Collection (Manassas, VA) were grown in Dulbecco’s Modified Eagle Medium (DMEM) supplemented with 10% fetal bovine serum, 2 mM l-glutamine, 100 units/ml penicillin and 100 μg/ml streptomycin (complete medium) at 37 °C.

### TUNEL assay

The extent of apoptosis was evaluated in cryostat liver tissue sections and Huh-7 cells using the terminal deoxynucleotidyl transferase-mediated dUTP-biotin nick end labeling (TUNEL) staining kit according to manufacturer’s instruction (Promega, Madison, WI). For TUNEL assay in cells, Huh-7 cells were cultured on glass coverslips in 6-well plates in complete medium up to 80% confluency. The cells were treated with or without, LPS (1 µg/ml) for 12 h in the presence or absence of Fc-apelin-13 (1 µM) and then fixed in 4% paraformaldehyde for 30 min. The fixed cells on cover slides were then processed for TUNEL assay. Nuclei were stained by 4′6-diamidino-2-phenylindole (DAPI) dye (1:800 dilution, Sigma). The number of TUNEL-positive nuclei was averaged over four randomly selected fields per section.

### Dihydroethidium (DHE) staining

DHE is a lipophilic cell-permeable dye that can undergo oxidation to ethidium bromide or a structurally similar product in the presence of superoxide and, to a lesser extent, hydrogen peroxide and hydroxyl radicals. Ethidium then binds irreversibly to the double-stranded DNA, causing amplification of a red fluorescent signal, and appears as punctate nuclear staining indicating ROS production^26^. To measure *in situ* ROS levels, frozen liver sections were incubated with DHE (5 μM, Invitrogen) at 37 °C for 30 min. For Huh-7 cells, the cells growing in the complete medium were pre-treated with Fc-apelin (1 μM) for 30 minutes and then LPS (1 μg/ml) for 1 hour before addition of DHE (5 μM) at 37 °C for 20 min, in wrapped aluminum foil. Three non-overlapping fluorescent images were obtained per slide with a microscope (Olympus IX-51) and staining density was quantified and averaged by ImageJ software (Bethesda, MD).

### Immunostaining and histology studies

Liver tissue samples were fixed in 10% formaldehyde for 24 hours and stored in PBS. The fixed tissues were processed routinely, embedded in paraffin, cut into 5 μm thickness and stained with hematoxylin and eosin (H&E). For immunohistochemistry (IHC), the tissue sections were subjected to deparaffination, 3% H_2_O_2_ treatment and antigen retrieval, and then primary antibody F4/80 (clone BM8, eBioscience, San Diego, CA) and HRP-conjugated goat anti-rabbit IgG or goat anti-mouse IgG (KPL, Gaithersburg, MD). Staining was developed with Vectastain ABC kit (Vector Laboratories, Burlingame, CA) and nuclei were stained by hematoxylin. Three non-overlapping areas were obtained and averaged per slide (x 200 magnification) with a microscope (Olympus IX-51).

### Quantitative real-time PCR analysis

Total RNAs were prepared from the snap-frozen tissue specimens using TRIzol (Invitrogen), and reverse transcription was carried out in a reaction containing 1 μg of total RNA, poly(dT) primer, and Moloney murine leukemia virus reverse transcriptase using a first-strand cDNA synthesis kit (Promega). Quantitative real-time PCR (qRT-PCR) was conducted on a LightCycler480 using the LightCycler 480 SYBR Green I Master Mix (Roche Diagnostics). The amplification protocol was as follows: 95 °C/5 minutes and 45 × (95 °C/10 second, 60 °C/20 second, and 72 °C/30 second). Following amplification, a dissociation curve analysis was performed to ensure purity of the PCR product. Primers for TNFα and IL-6 were described previously^27^. Beta-actin mRNA was used for normalization of cDNA loading.

### Data analysis

All data are presented as mean ± SEM. One-way analysis of variance (ANOVA) with Bonferroni post-hoc test was used for comparing group differences. Differences were considered statistically significant at the level of P < 0.05.

## Results

### Effects of Fc-apelin on serum ALT and IL-6

**Figure 1:**
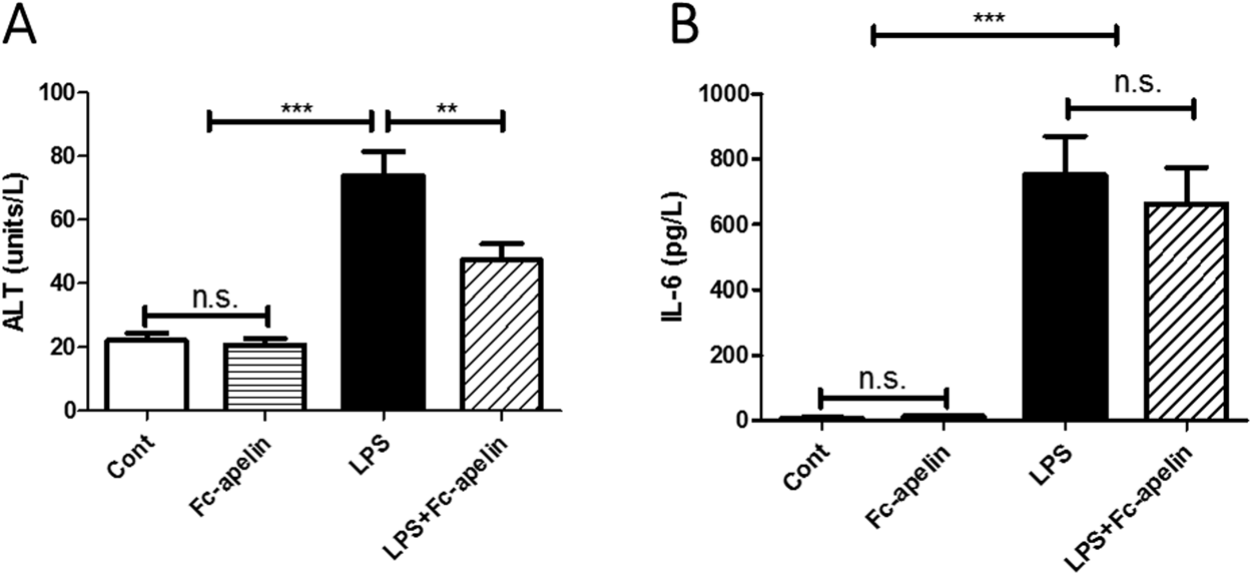
Effect of Fc-apelin on serum ALT and IL-6 in LPS-treated mice. (**A**) Effect on ALT (**B**) Effect on IL-6. Mice were administered with PBS (Cont) or Fc-apelin (1 mg/kg) with or without LPS (1 mg/kg) daily for five days. Data are expressed as mean + SE; ***P < 0.001 (*n* = 9 mice/group).

The effect of Fc-apelin on LPS-induced inflammatory liver injury was evaluated by measuring ALT levels in serum. As shown in Fig. 1A, compared with the control group, LPS administration significantly increased serum ALT levels by 3.3 fold from 22.2 to 73.8 unit/L (*p* < 0.001). Co-treatment with Fc-apelin significantly attenuated the LPS-induced increase in ALT activities, limiting the increase to 47.5 unit/L or 2.1 fold (*p* < 0.01), though the ALT levels were still higher than the control group. Fc-apelin alone had no effect on ALT in mice. Next, we assessed possible systemic effects of Fc-apelin on inflammation by measuring proinflammatory cytokine IL-6 levels in circulation (Fig. 1B). LPS greatly increased IL-6 with significant individual variations. Although Fc-apelin treatment appeared to decrease IL-6 levels, this difference was not significant. These data suggest that Fc-apelin may be protective against LPS-induced liver injury, without affecting systemic inflammation status.

### Fc-apelin is protective against LPS-induced apoptosis

**Figure 2:**
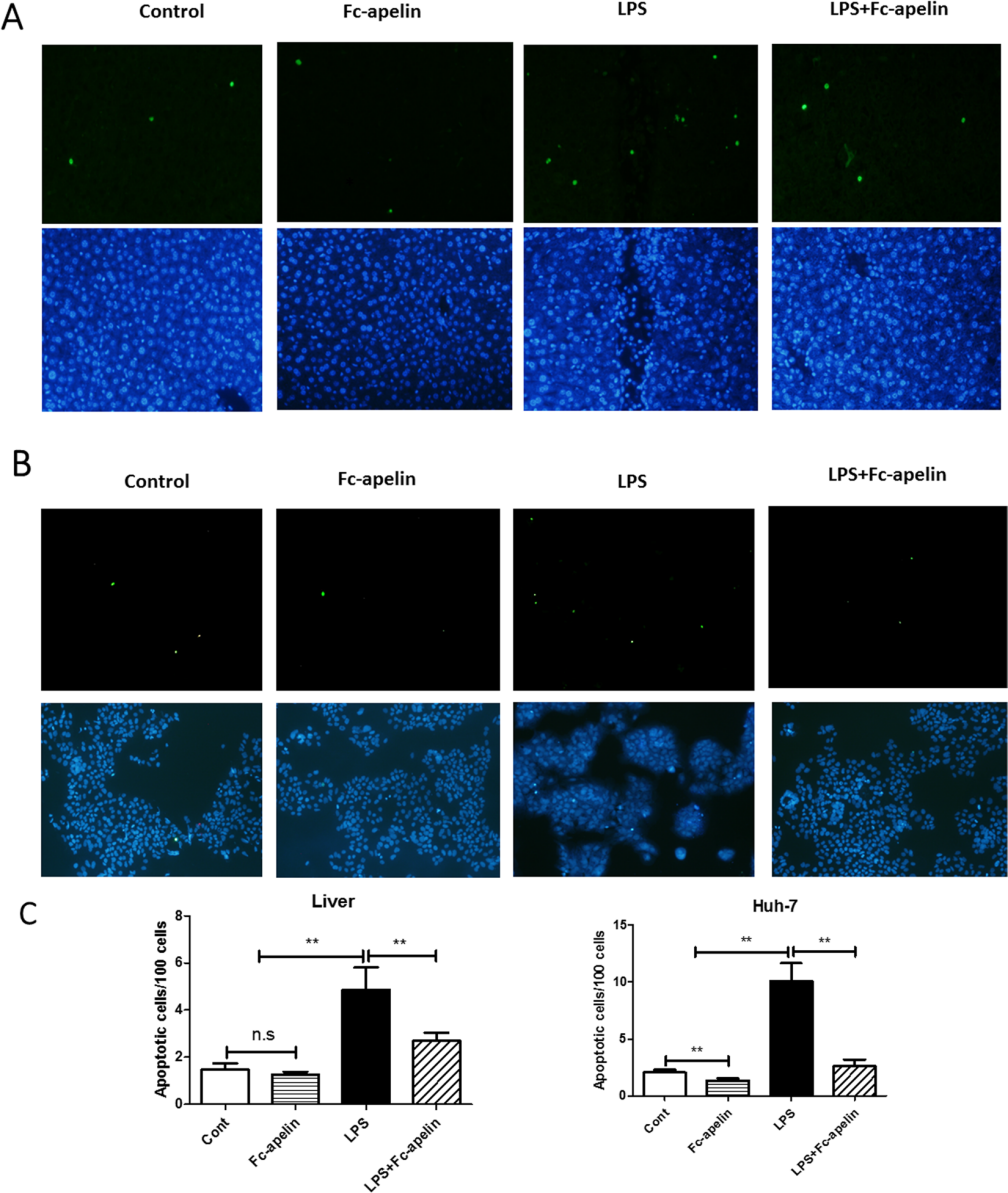
Fc-apelin prevents LPS-induced apoptosis. TUNEL assay was used to assess apoptosis of liver tissue sections (**A**) of mice receiving PBS, Fc-apelin (1 mg/kg) and/or LPS were assessed by TUNEL assay for apoptosis or Huh-7 cells (**B**) which were treated with PBS/albumin (Cont) or Fc-apelin (0.5 μM) with or without LPS (1 μg/ml) for 12 hours. Green staining indicates apoptotic cells and DAPI staining was used to visualize nuclei (low panel). The representative images are presented and data (**C**) are expressed as mean + SE; n = 5; ***p < 0.001and n.s.: no significance.

Hepatocyte apoptosis is an important early event underlying acute liver damage^28^. We therefore measured apoptosis in the liver. As depicted in Fig. 2, TUNEL staining indicated that hepatic apoptosis was increased by about 1.6 fold in mice receiving LPS, which was significantly attenuated by Fc-apelin treatment to levels near the control group. As controls, few apoptotic cells were observed in mice receiving PBS or Fc-apelin alone. To further determine whether Fc-apelin had anti-apoptotic effects at the cellular level, we treated human hepatoma Huh-7 cells with LPS and/or Fc-apelin and found that Fc-apelin could ameliorate LPS-induced apoptosis. Thus, Fc-apelin was protective against LPS-induced hepatic apoptosis and exerted direct anti-apoptotic effects on cultured hepatocytes *in vitro*.

### Reduction of LPS-induced ROS production by Fc-apelin

**Figure 3:**
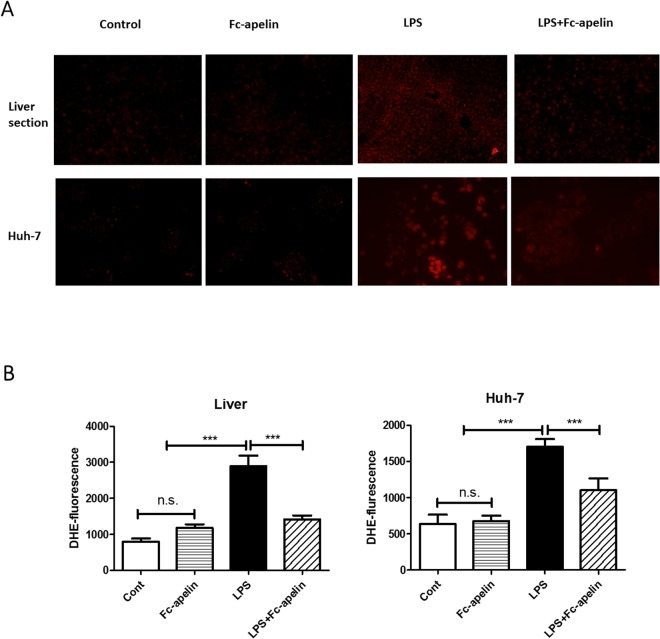
Detection of ROS in mouse livers by DHE staining. (**A**, upper panel) Liver tissue sections of mice receiving PBS, Fc-apelin (1 mg/kg) and/or LPS were stained with DHE and the fluorescence intensity was quantified by fluorescence microscope. (**A**, lower panel) Huh-7 cells were pre-treated with PBS/albumin (Cont) or Fc-apelin (0.5 μM) for 30 min and then with or without LPS (1 μg/ml) for one hour. The cells were incubated with DHE for 20 min and quantified by microscopy. (**B**) Quantification of ROS production was done by measuring fluorescence intensity by Image J. Data are expressed as mean + SE; n = 5; ***p < 0.001and n.s.: no significance.

ROS production is an initiator of hepatic inflammation by LPS and apelin has been found to be anti-oxidative. To determine whether the protective effect of Fc-apelin might be attributable to its anti-oxidative activities, we determined ROS levels in liver tissue sections and cultured hepatocytes by DHE fluorescence. As shown in Fig. 3, compared with the control group, mice injected with LPS showed a significant increase (*P* < 0.01) in the liver DHE staining. Co-treatment with Fc-apelin markedly lowered the DHE intensity. In contrast, PBS or Fc-apelin alone had no effect on ROS production (Fig. 3). In cultured Huh-7 cells, LPS also induced ROS production, which was ameliorated by Fc-apelin (Fig. 3). Thus, Fc-apelin suppressed LPS-induced ROS production both *in vivo* and *in vitro*.

### Effects of Fc-apelin on LPS-induced liver injury and macrophage infiltration

**Figure 4:**
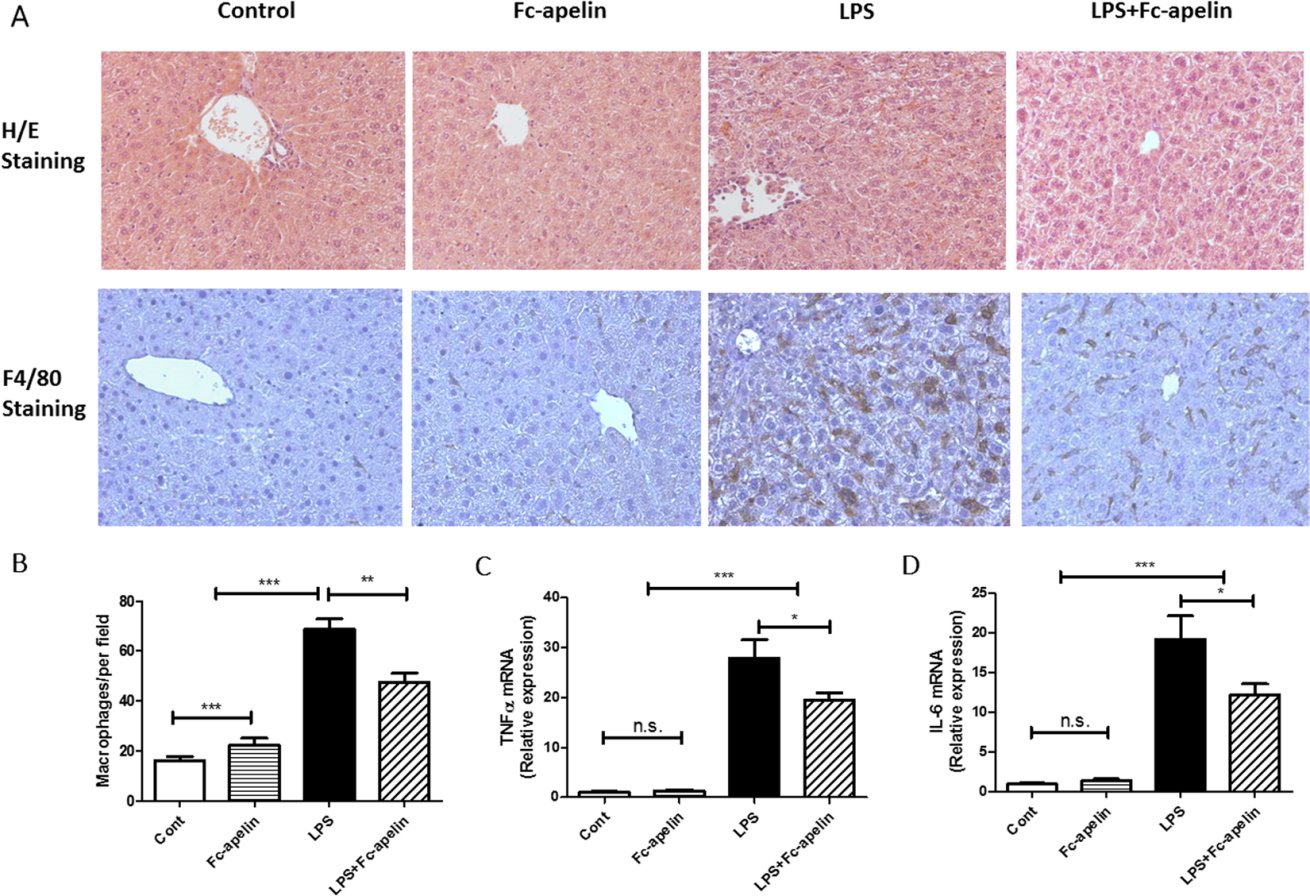
Fc-apelin ameliorates LPS-induced liver damage and inflammation responses. (**A**) Representative H/E staining and immunohistochemistry of macrophage marker F4/80 of liver sections. (**B**) Quantification of F4/80 + cells per view (200 x magnification). Quantitative PCR (qPCR) of gene expression for TNFα (**C**) and IL-6 (**D**). Gene expression is normalized with β-actin. Data are expressed as mean + SE; n = 5 for microphage infiltration analyses and n = 8 for qPCR; *p < 0.05; **p < 0.01; ***p < 0.001 and n.s.: no significance.

Macrophages play a pivotal role in pathogenesis of LPS-induced liver damage^29,30^. We next conducted histological studies to examine the effect of Fc-apelin on liver histology. In H/E staining (Fig. 4A), liver morphology appeared normal in mice receiving PBS and Fc-apelin. However, in the LPS-treated mice, focal necrosis and tissue structure distortion were apparent, which was significantly attenuated by co-treatment of Fc-apelin. The effect of Fc-apelin on macrophage infiltration was measured by IHC by using F4/80, a macrophage-specific marker^31,32^. As shown in Fig. 4B, LPS remarkably increased the macrophage labeling, which was substantially blunted by Fc-apelin treatment. Nevertheless, the extent of macrophage staining in the LPS + Fc-apelin group was still higher than the PBS or Fc-apelin-treated group. Thus, the apelin fusion protein can reduce liver injury and diminish macrophage infiltration induced by LPS.

### Effects of Fc-apelin on mRNA expression of TNFα and IL-6

Given the pronounced changes of the macrophages in the liver of mice receiving LPS and Fc-apelin treatment, we next measured mRNA expression of tumor necrosis factor-alpha (TNFα) and IL-6 in the liver. Figure 4B shows that the administration of LPS induced increases in TNF-α and IL-6 by about 28 and 19-fold, respectively, compared to PBS or Fc-apelin controls. The increase was attenuated by about 30 and 36%, respectively, by co-administration of Fc-apelin.

## Discussion

Besides its prominent inotropic activity and cell protective activities, apelin has been demonstrated to possess a broad range of anti-inflammatory properties from suppression of the NF-κB pathway and pro-inflammatory cytokines to anti-oxidation. Thus, apelin appears to be a potential therapeutic for endotoxemia-induced tissue injuries at multiple levels. However, the short *in vivo* half-life of apelin would limit its potential clinic application.

In the present study, we show a protective role of the long-acting Fc-apelin fusion protein^22^ against LPS-induced liver injury. The administration of LPS induced serum ALT elevation, indicating a liver damage. LPS-induced liver injury develops in a step-wise, but complex fashion involving activation of NF-κB, subsequent production of a variety of inflammatory mediators^33,34^, activation of liver macrophages (Kupffer cells), infiltration of blood-derived macrophages and neutrophils, and over-generation of ROS. This excessive inflammation leads to cell death and tissue destruction. In this study, we treated mice with relatively a low dose of LPS for 5 days and thus established a sub-acute model of endotoxemia in which multiple inflammatory events occur concurrently.

Apelin is a peptide hormone acting on APJ which is expressed in multiple tissues including the liver^35^ and in hepatoma cells^36^. Many studies have shown apelin’s protective activities against infection through anti-inflammation and promoting cell survival. For example, apelin reduces inflammatory cytokines in cerulein-induced chronic pancreatitis, partially through suppression of NF-κB signaling^37^. Luo *et al*.^38^. report that apelin suppresses the inflammatory response and promotes cell survival partially via activating the PI3K and AKT pathways in a mouse model of postburn sepsis. Moreover, apelin can directly reduce gene expression and secretion of inflammatory cytokines in Raw276.4 monocytes^39^, and lower serum inflammatory cytokines TNFα and IL-6. In this study, we basically reproduced those anti-inflammation effects observed in animals by apelin peptide. We demonstrated that administration of Fc-apelin fusion protein attenuated the apoptosis and ROS production both in cultured hepatoma cells and in mice challenged by LPS, suggesting that the fusion protein may exert both direct and indirect protective effects against LPS-inflicted cell damage. Notably, we found that Fc-apelin greatly reduced macrophage infiltration and liver tissue injury by histology, which is in agreement with the observed reduction of serum ALT. However, although we observed a decreased expression of inflammation markers of IL-6 and TNFα in the liver, we did not see a statistically significant decrease of systemic IL-6 levels by Fc-apelin treatment in LPS mice, which is not in line with the report where systemic levels of IL-6, TNFα and IL-1β are reduced by apelin peptide infusion in a sepsis model of rats^38^. The difference might be attributable to the model of inflammation and/or apelin dose.

It is worth noting that apelin’s protective effects against inflammation are not restricted to the liver. Activation of apelinergic system by apelin and ELA, a new ligand of APJ^40–42^ have been shown to improve survival and exert strong protective effects against cardiac and renal dysfunction in rats with sepsis^43–45^. Combining cardiac and renal functional benefits with anti-inflammatory activities in the liver makes the activation of the apelinergic system an appealing therapeutic approach for systemic inflammation. Our long-acting Fc-apelin is potentially a novel biologic for this disease condition. More studies are warranted to investigate the Fc-apelin’s protective activities in other inflammation animal models.
