# Author Correction: Fc-apelin fusion protein attenuates lipopolysaccharide-induced liver injury in mice

**DOI:** 10.1038/s41598-018-36258-7

**Published:** 2018-11-29

**Authors:** Huifen Zhou, Rongze Yang, Weimin Wang, Feng Xu, Yue Xi, Robert A. Brown, Hong Zhang, Lin Shi, Dalong Zhu, Da-Wei Gong

**Affiliations:** 1 Department of Pathology, Hubei University of Science and Technology, Xianning, Hubei 437100 China; 2 Division of Endocrinology, Diabetes and Nutrition, University of Maryland School of Medicine, Baltimore, USA; 3 Nanjing University School of Medicine, Nanjing, China

Correction to: *Scientific Reports* 10.1038/s41598-018-29491-7, published online 30 July 2018

The Acknowledgements section in this Article is incomplete.

“Partially supported by Maryland Stem Cell Research Fund”.

should read:

“Partially supported by Maryland Stem Cell Research Fund. The work is also partially supported by Science & Technology Project No. B2017179 from Hubei Provincial Department of Education to H.Z.”

